# Supplementary material for: Patterns of Intron Gain and Loss in Fungi
Source: PLoS Biol. 2004 Nov 30;2(12):e422. doi: 10.1371/journal.pbio.0020422 (PMC532390; doi:10.1371/journal.pbio.0020422)
Supplement: Table S1 — Also available at http://genes.mit.edu/NielsenEtAl/. (4.3 MB ZIP). [file pbio.0020422.st001.zip › NielsenEtAl/html/1216.html]

AN7360.1.NCU00465.1.MG05249.1.FG04121.1


```
 CLUSTAL W (1.82) Multiple Sequence Alignments - Introns Inserted


Sequence 1: NCU00465.1	423 aa
Sequence 2: FG04121.1	433 aa
Sequence 3: MG05249.1	443 aa
Sequence 4: AN7360.1	553 aa
Alignment Length: 601 aa
Number Identitical Residues: 156 aa
Alignment Score (without introns) 9226


MG05249.1 	MDGTNSFGGASPPNVDEDIDLYE1LLEIDRSASASDIKKAYHK0AARQHHPDKVPEDRKE
NCU00465.1	------MSSSSGD--EGDLDLYA1ILGVDKSASPNDIKKAYRK0LALIHHPDKVPEDQRP
FG04121.1 	MD--DPFNAGSPG--PEEVDLYE1LLEIDRTATPDQIKKAYRK0AALKYHPDKVAEEQRE
AN7360.1  	-----------------------~------MKSEKPTAKYGPD0AALANHPDKFPEEERD
          	                                : .   *   .  *   ****..*:.: 

MG05249.1 	EAEATFKAIQQAYEILRDDDKRHLYDTHGMAAFDPSRGPAGHGGMGGMGEDDLQDLFASM
NCU00465.1	EAEVKFKAIAQAYEILSDEEKREMYDVHGMAAFDPSRG-----GGHGAHGANMDDIFAAM
FG04121.1 	ESEAKFKEVTQAYEILSDEQKRELYDVHGMAAFDKSRG-----GPGGPE-VDLNDILSQM
AN7360.1  	AASVRFQEVQQAYEILSDDDKRHLYDTHGMGAFNGSGE------PGMNGGPDLDDILAQM
          	 :.. *: : ****** *::**.:**.***.**: *               :::*::: *

MG05249.1 	FMGGGMGGMG-GMGGMGGMGGRGGPRRPQRGADQEIPHEVTLEELYRGKTVKFASSKREL
NCU00465.1	F------GMG----GMGGMG--GMPRRPKRSPDEEQPYKVTLEELYKGKTVKFAAEKQVV
FG04121.1 	F------GFGMGPGGPGGPGGPGGPRRPRRGPDEEQEYKVTLEELYRGKTVKFAANKQVL
AN7360.1  	F------GMG----GMGGMPGMGGMPRPRQSPDEEQKYEVSLEELYRGKTVKFASTKNVI
          	*      *:*    * **  . *   **::..*:*  ::*:*****:*******: *. :

MG05249.1 	CAVCKGSGAREKYKPQDCDKCQASGFRRVLKQVGPGLVSQDVVECDHCHGTGKYVKEKER
NCU00465.1	CRQCKGTGAKENVKPNKCERCRGRGLVEAYQSIGPNMARQVVIPCDHCSGSGMHYKEKDR
FG04121.1 	CGQCKGSGGKEKAKAASCERCKGNGIVEAFRQIGPGMMRRETVLCDHCQGAGQVFKEKDR
AN7360.1  	CSLCKGKGGKEKATAKKCSTCDGHGFKEVLTRMG-QFLTKSTQTCTTCNGDGQFFATKDK
          	*  ***.*.:*: .. .*. * . *: ..   :*  :  : .  *  * * *     *::

MG05249.1 	CKKCKGKTTLPVTKALEIYIPRGAF~HGEKVVLEGEGDQLPGQTPGDIIIVLVEKSHDTF
NCU00465.1	CKKCKGKRTCKETKALELYIPPGSM~QGDRIVLEGEADQLPDQAPGDLIFHLVEEPHDVF
FG04121.1 	CKKCKGKRTSQEKKVLEIYIPRGSM~QGERIVLEGEADQHPDQTPGDIVFTLVEEPHDVF
AN7360.1  	CKKCKGKKTTEERKILEIYIPRGAK2DGDRIILEGEADQVPGQEPGDIIFQLEEEKHPVF
          	******* *    * **:*** *:  .*::::****.** *.* ***::: * *: * .*

MG05249.1 	TRIGHDLSAELNVTLAEALMGFSRVVLKHLDGRGLHLELPR---GKVLRPTEILKVPGEG
NCU00465.1	TRIGHDLSADLNVALIEALSGFSRVVVKHLDGRGIHINHPR---GKVLRPGDVLKVPGEG
FG04121.1 	TRIGHDLSAELTVTLGEALSGFSRTVFKHLDGRGIHIERPR---GKILRPGDCLKIPGEG
AN7360.1  	TRAGADLKATIDITLAESLTGFSRVVLKHLDGRGIELAHPKPGHSQILSPGQVLKVPGEG
          	** * **.* : ::* *:* ****.*.*******:.:  *:.. .::* * : **:****

MG05249.1 	MPHKRGDAKGDLYLVVKIEFPEDGWLKDGSDYEALQKLLPPPAAPITADEVDDVEYERDA
NCU00465.1	MPVKKSDMKGDLYLVVKIEFPEDGWLQDDSQYDALAKLLPPPPKPIEAEEIDDVEYESGA
FG04121.1 	MPMKRGEAKGDLYLIVKVEFPEDGWLKEDSEHDALQKMLPTAPANVEAEEVDEVEYEDDA
AN7360.1  	MPIKRSDARGDLYLIVNVKFPDENWKPSPAVLERLKEMLPKPGPQIQADTVDEVEYDPKG
          	** *:.: :*****:*:::**::.*  . :  : * ::** .   : *: :*:***:  .

MG05249.1 	DIEQ0MGASAGE-QGGSEWEDEEE-------~--AG------------------------
NCU00465.1	DIQE0MGAHQGDPRYGNDWEDDDED------~EGAG------------------------
FG04121.1 	DIEK0MGENSGDGRFGGDWEDDDDD------~MGDGQ-----------------------
AN7360.1  	NVEE~FG--SKDPSGGDAWEDDDEEGEGAQY1RGTGSRILTTPYNSTPLYTAISTTGINQ
          	:::: :*    :   *. ***:::....:.   . *.   ::. .::.  :: :::. ..

MG05249.1 	----------PQCATQ--------------------------------------------
NCU00465.1	----------PQCATQ--------------------------------------------
FG04121.1 	----------AQCQTQ--------------------------------------------
AN7360.1  	YQPSQDIPIMRRCSTSNGSILPGYRIRTHRNPAYNHKQSDSTRAPTRLLEDIEQESASDY
          	 ..:.. .   :* *...:  ..    :  ..: . ..:.:: :.:   .. ...:::. 

MG05249.1 	----------------~-------------------------------------------
NCU00465.1	----------------~-------------------------------------------
FG04121.1 	----------------~-------------------------------------------
AN7360.1  	QTKSPSFNQTLFSQNR0CPQAQILSQNVEFRKRTFAPKTVPVRKQTLFTDDPDLLVCDVL
          	.:.:.: ..:  :..  ...:.  :.. .  . : :..: .  ..:  :....   ..  

MG05249.1 	-------
NCU00465.1	-------
FG04121.1 	-------
AN7360.1  	KEEKSAQ
          	....::.
```
